# Clinical Characteristics and Prognosis of Metaplastic Breast Cancer Compared with Invasive Ductal Carcinoma: A Propensity-Matched Analysis

**DOI:** 10.3390/cancers15051556

**Published:** 2023-03-02

**Authors:** Jun-Hee Lee, Jai Min Ryu, Se Kyung Lee, Byung Joo Chae, Jeong Eon Lee, Seok Won Kim, Seok Jin Nam, Jonghan Yu

**Affiliations:** Division of Breast Surgery, Department of Surgery, Samsung Medical Center, School of Medicine, Sungkyunkwan University, 81 Irwon-ro, Gangnam-gu, Seoul 06351, Republic of Korea

**Keywords:** metaplastic breast cancer, invasive ductal carcinoma, triple negative breast neoplasm, propensity score, survival

## Abstract

**Simple Summary:**

Metaplastic breast cancer (MpBC) is a rare disease—an aggressive subtype among breast cancers. Though there are no clear treatment guidelines, MpBC generally is treated aggressively by chemotherapy or radiation therapy because of its rarity and propensity to recur. We assessed the clinical characteristics and prognosis of MpBC in real world data to better understand the MpBC disease entity and perform the appropriate treatment.

**Abstract:**

Background: Metaplastic breast cancer (MpBC) is an aggressive histologic type of breast cancer. Although MpBC has a poor prognosis and is responsible for a large proportion of breast cancer mortalities, the clinical features of MpBC compared with invasive ductal carcinoma (IDC) are not well known, and the optimal treatment has not been identified. Methods: We retrospectively reviewed medical records of 155 MpBC patients and 16,251 IDC cases who underwent breast cancer surgery in a single institution between January 1994 and December 2019. The two groups were matched 1:4 by age, tumor size, nodal status, hormonal receptor status, and HER2 status using propensity-score matching (PSM). Finally, 120 MpBC patients were matched with 478 IDC patients. Disease-free survival and overall survival of MpBC and IDC patients both before and after PSM were analyzed by Kaplan-Meier survival, and multivariable Cox regression analysis was performed to identify variables affecting long-term prognosis. Results: The most common subtype of MpBC was triple-negative breast cancer, and nuclear and histologic grades were higher than those of IDC. Pathologic nodal staging of the metaplastic group was significantly lower than that of the ductal group, and more frequent adjuvant chemotherapy was performed in the metaplastic group. Multivariable Cox regression analysis indicated that MpBC was an independent prognostic factor for disease-free survival (HR = 2.240; 95% CI, 1.476–3.399, *p* = 0.0002) and overall survival (HR = 1.969; 95% CI, 1.147–3.382, *p* = 0.0140). However, survival analysis revealed no significant difference between MpBC and IDC patients in disease-free survival (HR = 1.465; 95% CI, 0.882–2.432, *p* = 0.1398) or overall survival (hazard ratio (HR) = 1.542; 95% confidential interval (CI), 0.875–2.718, *p* = 0.1340) after PSM. Conclusion: Although the MpBC histologic type had poor prognostic factors compared with IDC, it can be treated according to the same principles as aggressive IDC.

## 1. Introduction

Female breast cancer was the most common cancer in 2020, surpassing lung cancer [1]. Nearly half of breast cancer cases are diagnosed in Asia [1]. Metaplastic breast cancer (MpBC) is a rare histologic subtype that accounts for only 0.2%–2% of breast cancers, and it has been found that there is a high incidence of MpBC diagnosis among the black race in some studies [2,3]. This cancer is comprised of poorly differentiated tumors and heterogeneous histology involving both mesenchymal and epithelial components of spindle cells, squamous differentiation, and chondroids [4,5,6]. Variants of MpBC include carcinosarcoma, matrix-producing carcinoma, sarcomatoid carcinoma, pseudosarcoma, and mixed tumors of the breast [1].

MpBC is a clinically aggressive breast cancer with poor prognosis [7,8]. Such tumors tend to be larger and have more node negativity than other types. In addition, it carries a greater risk of metastasis than other histologic subtypes, patients present with more advanced disease stage, and the cancer is less responsive to conventional adjuvant and neoadjuvant chemotherapy [9,10]. MpBC is characterized by chemoresistance and hematogenous spread, in contrast to the lymphatic dissemination typically seen with invasive ductal carcinoma (IDC) [4,11]. MpBC is more likely to be treated with mastectomy than breast-conserving surgery, and such patients have higher mastectomy rates than patients with IDC. Despite this higher rate, this type of surgery was not associated with a survival difference [2]. The molecular subtypes of MpBC are basal-like breast cancer with estrogen receptor negativity, progesterone receptor negativity, and human epidermal growth factor receptor 2 (HER2) negativity; and MpBC is more aggressive than triple negative breast cancer (TNBC), having a worse prognosis [12,13,14]. In addition, the TNBC subtype of MpBC is worse than that of IDC [15]. In addition, although many studies have analyzed MpBC, only one simple comparison with IDC was performed and did not adjust for prognostic factors of MpBC.

There are no treatment guidelines specific to the management of MpBC, which is frequently misdiagnosed or unrecognized on pathologic review because of its rarity and heterogeneity [4]. MpBC tends to be over-treated compared to typical IDC, even at a lower pathologic stage because of its aggressiveness [16]. However, there are few studies comparing clinical features and prognosis between MpBC and IDC because of the rarity of MpBC.

In the present study, we investigated the characteristics of MpBC in the real world and compared prognosis between MpBC and IDC using propensity-score matching to adjust confounding factors.

## 2. Patients and Methods

### 2.1. Study Population

We retrospectively reviewed the clinical data of 27,675 patients with breast cancer who were treated with surgery from 1994 to 2019 in the Samsung Medical Center.

We investigated extensive pathologic findings about histopathology, multiplicity, lymphatic invasion, extensive intraductal component, nuclear grade, and histologic grade. We reviewed molecular pathologic markers of estrogen receptor status, progesterone receptor status, and HER2 status. We also investigated patients who underwent chemotherapy, radiation therapy, or hormone therapy instead of surgery.

### 2.2. Propensity-Score Matching

Propensity-score matching (PSM) is a method for filtering experimental and control cases of similar characteristics, which are called the matching variables, for comparison in a retrospective analysis [17]. PSM can be used to adjust for baseline characteristics and reduce the effect of selection bias. The variables for PSM in this study were age (years), histopathology, multiplicity, lymphatic invasive, extensive intraductal component, nuclear and histologic grade, tumor size, nodal status, estrogen receptor, progesterone receptor, HER2, hormonal therapy, chemotherapy, and radiation therapy. We used a ratio of 1:4 for nearest neighbor matching within 0.2 standard deviations of the logit of the propensity score. The propensity score was analyzed using logistic regression. We assessed the balance of covariates in the two groups after PSM, the results of which are shown in Supplementary Table S1.

### 2.3. Statistical Analyses

Patient characteristics were compared using the independent *t*-test for continuous variables and the chi-square or Fisher’s exact test for categorical variables. Univariable and multivariable analyses were conducted using Cox regression analysis models. After excluding patients due to missing values, Cox regression analysis models were tested on 12,905 out of 16,406 total patients. Statistical significance was established at *p* < 0.05. All statistical analyses were performed using the Statistical Analysis System (SAS) version 9.4 (SAS Institute Inc., Cary, NC, USA).

## 3. Results

**Figure 1:**
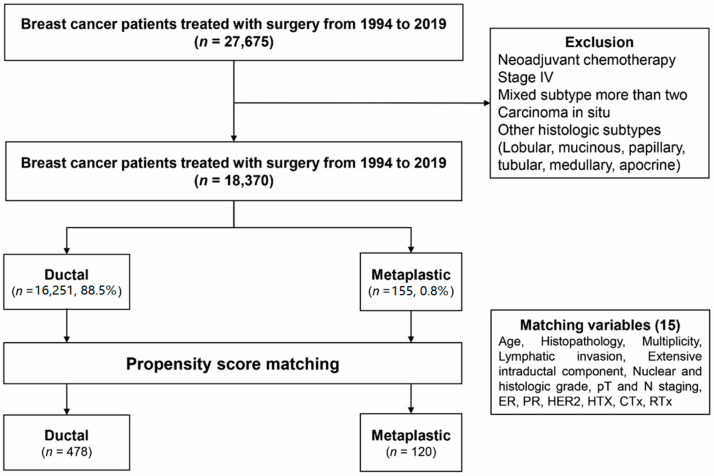
Consort diagram of this study.

We investigated 27,675 breast cancer patients treated with surgery from 1994 to 2019 at the Samsung Medical Center. We excluded patients with stage IV cancer or carcinoma in the breast at the time of diagnosis and patients receiving neoadjuvant chemotherapy. We also excluded patients confirmed with a mixed tumor subtype greater than grade two on final pathology to remove confounding variables and analyze by pure subtype. Among 18,370 cases of invasive carcinoma, the ductal type comprised 88.5% (16,251 patients), and the metaplastic type was 0.8% (155 patients); other types, such as lobular, mucinous, and papillary, accounted for 10.8% of the population (1964 patients). After excluding patients with missing values, the completed set was analyzed during propensity-score matching. Finally, after 1:4 propensity-score matching, 478 patients of ductal type and 120 patients of metaplastic type were compared (Figure 1).

Demographics and clinical characteristics before PSM are shown in Table 1. There was no difference in age between the two groups, but there were differences in all other variables. Multiplicity, lymphatic invasion, and extensive intraductal component (EIC) were significantly higher in the ductal group than the metaplastic group. Nuclear and histologic grade and tumor size were significantly higher in the metaplastic group than the ductal group. In the metaplastic group, the proportion of patients with estrogen receptor positivity was about 10%, and more than 95% of cases were negative for progesterone receptor and HER2. About 60% of the ductal group received adjuvant chemotherapy, compared with more than 90% of the metaplastic group. After 1:4 PSM, there was no difference in any variables except progesterone receptor status (Supplementary Table S2).

**Figure 2:**
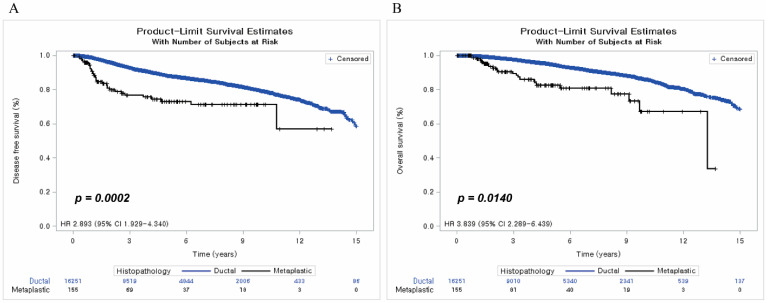
Disease-free survival and overall survival before matching. (**A**) Disease-free survival before matching; (**B**) Overall survival before matching.

The median follow-up duration of the overall population was 68 months (range 3–277 months). Disease-free survival (DFS) of the ductal group was 89.98%, and that of the metaplastic group was 78.06%, with a hazard ratio of 2.893 (95% confidence interval (CI): 1.929–4.340) before PSM (*p* = 0.0002). Overall survival (OS) of the ductal group was 94.04%, and that of the metaplastic group was 85.16%, with a hazard ratio of 3.839 (95% CI: 2.289–6.439) before PSM (*p* = 0.0140) (Figure 2).

**Figure 3:**
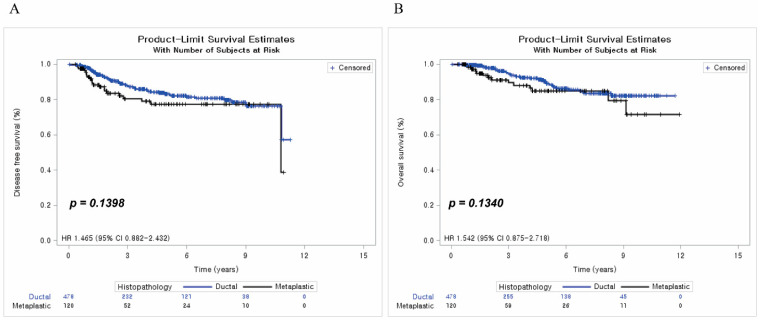
Disease-free survival and overall survival after matching. (**A**) Disease-free survival after matching; (**B**) Overall survival after matching.

After 1:4 PSM, the DFS of the ductal group was 87.45%, and that of the metaplastic group was 82.50%, with an HR of 1.465 (95% CI: 0.882–2.432) (*p* = 0.1398). The OS of the ductal group was 91.84%, and that of the metaplastic group was 88.33%, with an HR of 1.542 (95% CI: 0.875–2.718) (*p* = 0.1340) (Figure 3).

We analyzed the clinically significant prognostic factors using a Cox regression model for univariable and multivariable analyses. This analysis showed lymphatic invasion, histologic grade, pT and pN stage, ER, radiotherapy, and chemotherapy as significant factors for DFS and OS (Table 2, Table 3, Table 4 and Table 5). Inclusion in the metaplastic group was a significant, poor prognostic factor for DFS (HR = 2.240; 95% CI: 1.476–3.399, *p* = 0.0002) and OS (HR = 1.969; 95% CI: 1.147–3.382, *p* = 0.0140).

## 4. Discussion

As shown in previous studies, metaplastic breast cancer has more aggressive features than other types [1,2]. Patients with MpBC present with a higher histologic grade, with less nodal involvement, and at an advanced stage. Due to the advanced stage, their prognosis is poorer than that of IDC. However, after performing PSM to correct for variables that affect a tumor, there were no differences in disease-free and overall survival between MpBC and IDC groups.

MpBC is a rare disease that is clinically characterized by a poor prognosis and has shorter disease-specific survival than IDC. Owing to this, when MpBC is diagnosed, there is a tendency toward aggressive treatment [2]. However, the intrinsic nature of MpBC might not significantly differ from that of IDC based on our propensity-score-matched data. It is possible that we did not have the correct general knowledge of the nature of MpBC because of a lack of comparative analysis of MpBC and IDC using PSM.

Although MpBC is a rare disease, the histological subtypes and genomic profiles of MpBC are diverse. Histologically, MpBC tumors are heterogeneous, poorly differentiated tumors with epithelial and mesenchymal components, including ductal carcinoma [4]. The epithelial types of MpBC are largely divided into squamous cell carcinoma, adenocarcinoma with spindle cell differentiation, and adenosquamous carcinoma. The mesenchymal types of MpBC consist of carcinoma with chondroid metaplasia, carcinoma with osseous metaplasia, and carcinosarcoma. Some molecular and genetic features of MpBC are known to be involved in its pathogenesis, including the epithelial–mesenchymal transition pathway; the mitogen-activated protein (MAP) kinase signaling pathway; the phosphoinositide 3-kinase (PI3K) pathway; and the epithelial growth factor pathways, including those of protein kinase B (Akt), mammalian target of rapamycin (mTOR), and epithelial growth factor receptor (EGFR).

MpBC shows aggressive clinical characteristics, including a larger tumor size than other subtypes and histologically high nuclear and tumor grades. Oberman reported that a tumor size less than 4 cm was associated with a good prognosis, and that tumor size at the time of diagnosis was correlated with prognosis [18]. MpBC mainly demonstrates hematogenous spread and is prone to metastasize to other organs, thereby having a poor prognosis. Additionally, patients with MpBC are generally over 60 years old, and those who have distant metastases and do not receive radiotherapy have worse prognoses in terms of disease-free survival and overall survival than patients with IDC. Thus, when MpBC is diagnosed, chemotherapy and radiotherapy generally are performed, along with surgical treatment. In this study, in the MpBC group, chemotherapy was performed in 90% of cases, in contrast to 60% in the ductal group.

MpBC is thought to be chemoresistant, even in a metastatic setting [5,19]. However, the diverse histological subtypes and genomic profiles of MpBC result in diverse response to chemotherapy. Yam C et al. reported a 23% pathologically complete response (pCR) rate in MpBC patients treated with neoadjuvant chemotherapy (NAC) and suggested that patients with MpBC receiving NAC should have careful monitoring of cancer progression because its pCR rate is lower than that of ductal TNBC [20]. In patients with clinically node-negative MpBC, NAC was linked to worse outcomes compared to adjuvant chemotherapy, but no such association was observed for those with clinically node-positive MpBC.

Thus, it is inaccurate to describe MpBC as chemoresistant because the responsiveness to such therapy should be evaluated considering various factors, such as tumor size, nodal status, nuclear and histologic grade, and Ki-67. Although MpBC can be resistant to chemotherapy, responsiveness and survival may differ by subtype. Schroeder et al. reported that survival of HER2-positive MpBC was improved and similar to that of HER2-positive IDC, and suggested that HER2-positive MpBC might be responsive to additional HER2-directed therapy [21]. When considering chemoresistance, the regimen should be assessed [22].

Through the development of immuno-oncology (IO), targeted therapy is in the spotlight for breast cancer. MpBC frequently involves tumor infiltrating lymphocytes (TILs) in the tumor microenvironment, sharing many features with the basal-like subtype [23,24]. Recent studies have demonstrated frequent overexpression of programmed death-ligand 1 (PD-L1) in MpBC, prompting interest in combining immune checkpoint inhibitors with conventional chemotherapy [25,26,27]. Use of IO for MpBC can be improved by identifying immune checkpoint inhibitor markers that increase expression in TIL.

Although systemic therapy is important for the treatment of MpBC, radical surgical treatment with sufficient margin should not be overlooked for locoregional control. Surgical treatment of MpBC is as successful as surgery for IDC [28]. In MpBC patients, mastectomy is performed at a higher rate than in IDC patients because of the larger tumor size [28]. In our study, the mastectomy rate was not high because there were many patients with pathologic T2 staging and few with pathologic T1 and T3 stages. However, no difference in disease-free survival or overall survival has been shown between mastectomy and lumpectomy [29]. The survival rate for MpBC compared to other types of breast cancer is not affected by the extent of surgical intervention. However, LI XIA et al. reported that overall survival was significantly higher in patients who received lumpectomy with radiation therapy compared to those who had a mastectomy [30]. MpBC generally demonstrates hematogenous rather than lymphatic metastases. Several studies have reported that MpBC patients had a lower rate of nodal involvement in the axillary area than IDC patients [28,29,31]. Tseng and Martinez reported 22% of MpBC patients had a metastatic axillary lymph node, and Pezzi et al. also reported 22% of MpBC patients had axillary lymph node involvement, in contrast with 34% of IDC patients [29,31]. Even if MpBC metastasizes less frequently to lymph nodes than does IDC, axillary lymph node surgery is important for local control and clear staging for adjuvant therapy. Although the rate of nodal involvement is low in MpBC, axillary nodal surgery is important because lymph-node metastases are present in about 20% of cases.

The main limitation of this study is that it did not present a difference in prognosis according to the subtype of MpBC because histologic grade, hormonal receptor status, and HER2 depend on subtype. Therefore, it is important to establish a molecular subtype and to target therapy or immunotherapy rather than basic treatment. Another limitation is that the study was conducted at a single institution with a retrospective design and was not a randomized controlled study (RCT). Despite these limitations, the large number of patients might allow a representative conclusion because MpBC is rare and RCT is difficult to perform in this type of study.

## 5. Conclusions

MpBC is an aggressive histologic subtype and has a poor prognosis compared with IDC. Based on PSM, there were no differences in disease-free and overall survival between MpBC and IDC. However, since there was a difference in tendency pf around 1.5 times in the hazard ratio, it is difficult to say that there is no complete difference. This suggests that treatment of MpBC should be in line with the treatment principles of the TNBC subtype of aggressive IDC. In the future, additional studies on the characteristics of MpBC, and new treatment modalities, such as IO, are expected to be applied.

## Figures and Tables

**Table 1:** Patient demographics and clinical characteristics before propensity-score matching.

	Metaplastic N (%)	Ductal N (%)	*p*-Value
Age (years)			0.8205
Mean	50.16	49.87	
Std	9.94	11.34	
Multiplicity			<0.0001
No	148 (95.48)	12,483 (76.81)	
Yes	7 (4.52)	3725 (22.92)	
Unknown	0 (0.00)	43 (0.27)	
Lymphatic invasion			0.0001
No	126 (81.29)	10,868 (66.88)	
Yes	23 (14.84)	4787 (29.46)	
Unknown	6 (3.87)	43 (3.66)	
EIC			<0.0001
No	130 (83.87)	10,506 (64.65)	
Yes	14 (9.03)	4866 (29.94)	
Unknown	11 (7.1)	879 (5.61)	
Nuclear grade			<0.0001
Low	1 (0.64)	1894 (11.65)	
Intermediate	21 (13.55)	8761 (53.91)	
High	129 (83.23)	5402 (33.24)	
Unknown	4 (2.58)	194 (1.20)	
Histologic grade			<0.0001
Good	4 (2.58)	3456 (21.27)	
Moderate	22 (14.19)	7443 (45.80)	
Poor	124 (80.00)	4957 (30.50)	
Unknown	5 (3.23)	395 (2.43)	
pT stage			<0.0001
T1	42 (27.10)	9882 (60.81)	
T2	102 (65.81)	5837 (35.92)	
T3	10 (6.45)	501 (3.08)	
T4	1 (0.65)	26 (0.16)	
Unknown	0 (0.00)	5 (0.03)	
pN stage			0.0002
N0	124 (80.00)	10,257 (63.12)	
N1	25 (16.13)	4275 (26.31)	
N2	5 (3.23)	1070 (6.58)	
N3	1 (0.65)	595 (3.66)	
Unknown	0 (0.00)	54 (0.33)	
Estrogen receptor			<0.0001
Negative	135 (87.10)	3751 (23.08)	
Positive	19 (12.26)	12,361 (76.06)	
Unknown	1 (0.64)	139 (0.86)	
Progesterone receptor			<0.0001
Negative	146 (94.19)	4972 (30.60)	
Positive	8 (5.16)	11,133 (68.51)	
Unknown	1 (0.65)	146 (0.89)	
HER2			<0.0001
Negative	142 (91.61)	12,079 (74.33)	
Positive	9 (5.81)	3475 (21.38)	
Unknown	4 (2.58)	697 (4.29)	
Hormone therapy			<0.0001
No	131 (84.52)	3736 (22.99)	
Yes	21 (13.55)	12,246 (75.36)	
Unknown	3 (1.93)	269 (1.65)	
Radiation therapy			0.0094
No	26 (16.77)	4267 (26.26)	
Yes	124 (80.00)	11,678 (71.86)	
Unknown	5 (3.23)	306 (1.88)	
Chemotherapy			<0.0001
No	11 (7.10)	5558 (34.20)	
Yes	140 (90.32)	10,435 (64.21)	
Unknown	4 (2.58)	258 (1.59)	

Abbreviations: EIC = extensive intraductal component; pT = pathologic T stage; pN = pathologic N stage; N = number of patients.

**Table 2:** Univariable analysis of disease-free survival (DFS).

DFS		Univariable Analysis	
(12,905 Patients)		HR (95% CI)	*p*-Value
Age (years)		0.982 (0.975–0.989)	<0.0001
Histology	Metaplastic vs. Ductal	2.893 (1.929–4.340)	<0.0001
Lymphatic invasion	Negative vs. Positive	0.498 (0.348–0.567)	<0.0001
Histologic grade	Moderate vs. Good	2.419 (1.866–3.136)	<0.0001
	Poor vs. Good	3.923 (3.039–5.064)	<0.0001
pT stage	pT2 vs. pT1	1.960 (1.665–2.309)	<0.0001
	pT3 vs. pT1	3.215 (2.350–4.400)	<0.0001
	pT4 vs. pT1	4.022 (1.208–13.388)	0.0168
pN stage	pN1 vs. pN0	1.365 (1.136–1.642)	0.0002
	pN2 vs. pN0	2.086 (1.609–2.704)	<0.0001
	pN3 vs. pN0	4.778 (3.704–6.164)	<0.0001
Estrogen receptor	Negative vs. Positive	1.923 (1.683–2.197)	<0.0001
Radiotherapy	Negative vs. Positive	1.556 (1.354–1.789)	<0.0001
Chemotherapy	Negative vs. Positive	0.642 (0.547–0.755)	<0.0001

Abbreviation: pT = pathologic T stage; pN = pathologic N stage.

**Table 3:** Multivariable analysis of disease-free survival (DFS).

DFS		Multivariable Analysis	
(12,905 Patients)		HR (95% CI)	*p*-Value
Age (years)		0.981 (0.975–0.988)	<0.0001
Histology	Metaplastic vs. Ductal	2.240 (1.476–3.399)	0.0002
Lymphatic invasion	Negative vs. Positive	0.718 (0.615–0.839)	<0.0001
Histologic grade	Moderate vs. Good	2.006 (1.531–2.627)	<0.0001
	Poor vs. Good	2.785 (2.071–3.745)	<0.0001
pT stage	pT2 vs. pT1	1.377 (1.145–1.655)	0.0001
	pT3 vs. pT1	1.751 (1.234–2.485)	0.0004
	pT4 vs. pT1	2.699 (0.800–9.108)	0.1524
pN stage	pN1 vs. pN0	1.218 (0.988–1.501)	0.0716
	pN2 vs. pN0	1.897 (1.405–2.553)	<0.0001
	pN3 vs. pN0	3.975 (2.898–5.451)	<0.0001
Estrogen receptor	Negative vs. Positive	1.553 (1.325–1.820)	<0.0001
Radiotherapy	Negative vs. Positive	1.815 (1.565–2.106)	<0.0001
Chemotherapy	Negative vs. Positive	1.797 (1.468–2.200)	<0.0001

Abbreviation: pT = pathologic T stage; pN = pathologic N stage.

**Table 4:** Univariable analysis of overall survival (OS).

OS		Univariable Analysis	
(12,905 Patients)		HR (95% CI)	*p*-Value
Age (years)		1.035 (1.025–1.045)	<0.0001
Histology	Metaplastic vs. Ductal	3.839 (2.289–6.439)	<0.0001
Lymphatic invasion	Negative vs. Positive	0.425 (0.350–0.514)	<0.0001
EIC	Negative vs. Positive	1.374 (1.106–1.708)	0.0042
Histologic grade	Moderate vs. Good	2.358 (1.545–3.600)	<0.0001
	Poor vs. Good	5.275 (3.518–7.910)	<0.0001
pT stage	pT2 vs. pT1	2.996 (2.321–3.869)	<0.0001
	pT3 vs. pT1	5.416 (3.552–8.256)	<0.0001
	pT4 vs. pT1	5.678 (1.032–31.225)	0.0443
pN stage	pN1 vs. pN0	2.053 (1.551–2.716)	<0.0001
	pN2 vs. pN0	3.216 (2.232–4.633)	<0.0001
	pN3 vs. pN0	7.272 (5.110–10.350)	<0.0001
Estrogen receptor	Negative vs. Positive	2.972 (2.455–3.598)	<0.0001
Radiotherapy	Negative vs. Positive	1.440 (1.167–1.777)	0.0007
Chemotherapy	Negative vs. Positive	0.706 (0.556–0.898)	0.0045

Abbreviation: EIC = extensive intraductal component; pT = pathologic T stage; pN = pathologic N stage.

**Table 5:** Multivariable analysis of overall survival (OS).

OS		Multivariable Analysis	
(12,905 Patients)		HR (95% CI)	*p*-Value
Age (years)		1.024 (1.014–1.034)	<0.0001
Histology	Metaplastic vs. Ductal	1.969 (1.147–3.382)	0.0140
Lymphatic invasion	Negative vs. Positive	0.662 (0.526–0.834)	0.0005
EIC	Negative vs. Positive	1.160 (0.923–1.457)	0.2028
Histologic grade	Moderate vs. Good	1.731 (1.114–2.689)	0.0105
	Poor vs. Good	2.710 (1.699–4.322)	<0.0001
pT stage	pT2 vs. pT1	1.823 (1.365–2.436)	<0.0001
	pT3 vs. pT1	2.947 (1.835–4.733)	<0.0001
	pT4 vs. pT1	1.535 (0.269–8.749)	1.0000
pN stage	pN1 vs. pN0	1.851 (1.355–2.531)	<0.0001
	pN2 vs. pN0	2.654 (1.744–4.039)	<0.0001
	pN3 vs. pN0	5.012 (3.223–7.793)	<0.0001
Estrogen receptor	Negative vs. Positive	2.437 (1.936–3.067)	<0.0001
Radiotherapy	Negative vs. Positive	1.636 (1.299–2.062)	<0.0001
Chemotherapy	Negative vs. Positive	2.576 (1.900–3.492)	<0.0001

Abbreviation: EIC = extensive intraductal component; pT = pathologic T stage; pN = pathologic N stage.

## Data Availability

The datasets used and/or analyzed during the study are available from the corresponding author, on reasonable request.

## Supplementary Materials

The following supporting information can be downloaded at: https://www.mdpi.com/article/10.3390/cancers15051556/s1. Table S1: Summary of balance table of propensity-score matching. Table S2: Patient demographic and clinical characteristics after propensity-score matching.
