# Supplementary material for: Clinical Characteristics and Prognosis of Metaplastic Breast Cancer Compared with Invasive Ductal Carcinoma: A Propensity-Matched Analysis
Source: Cancers (Basel). 2023 Mar 2;15(5):1556. doi: 10.3390/cancers15051556 (PMC10000576; doi:10.3390/cancers15051556)
Supplement: Supplementary file 1 [file cancers-15-01556-s001.zip › cancers-2169902-supplementary table S1.pptx]

## Slide 1
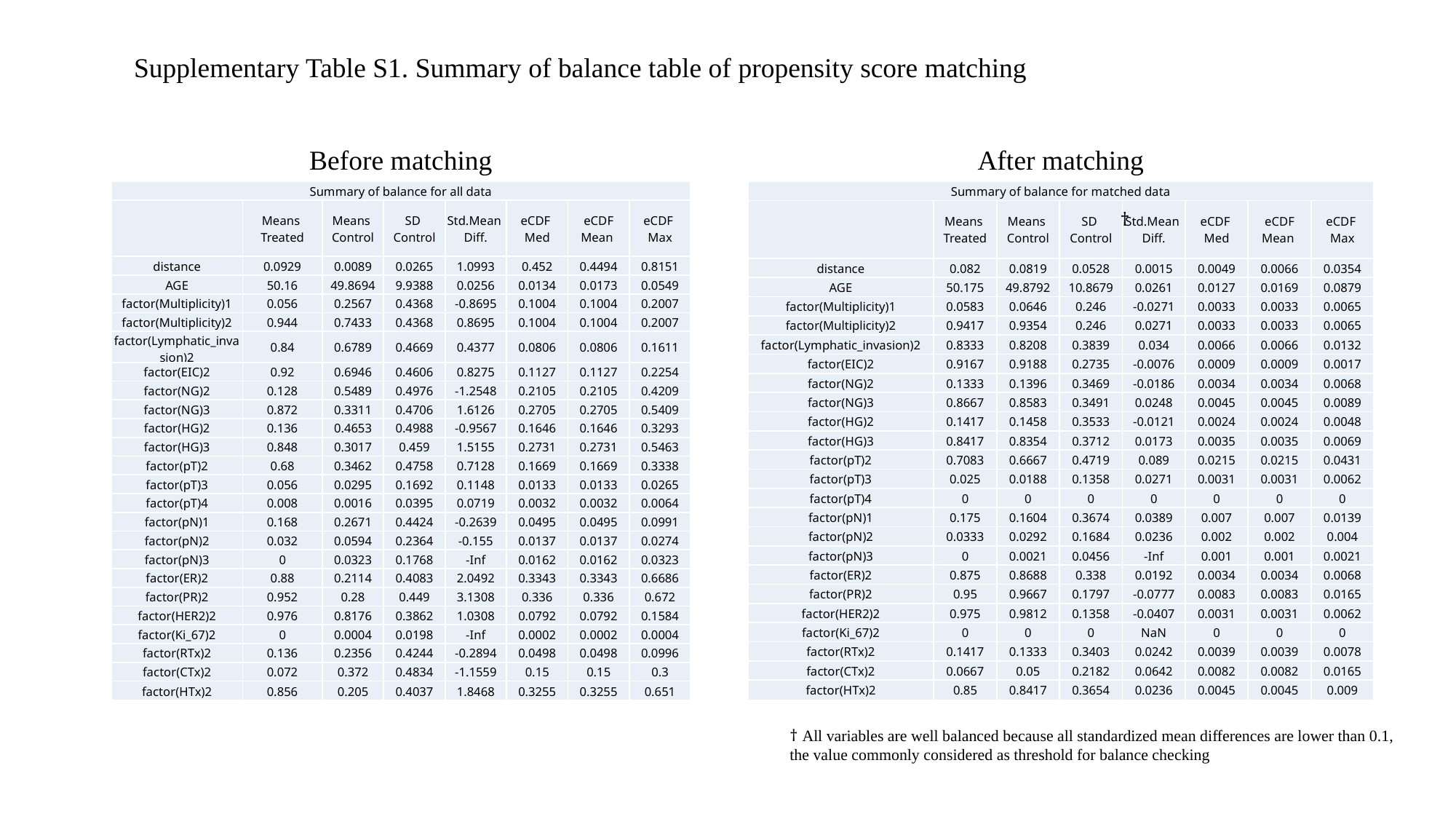

Supplementary Table S1. Summary of balance table of propensity score matching
Before matching
After matching
| Summary of balance for all data | | | | | | | |
| --- | --- | --- | --- | --- | --- | --- | --- |
| | Means Treated | Means Control | SD Control | Std.Mean Diff. | eCDF Med | eCDF Mean | eCDF Max |
| distance | 0.0929 | 0.0089 | 0.0265 | 1.0993 | 0.452 | 0.4494 | 0.8151 |
| AGE | 50.16 | 49.8694 | 9.9388 | 0.0256 | 0.0134 | 0.0173 | 0.0549 |
| factor(Multiplicity)1 | 0.056 | 0.2567 | 0.4368 | -0.8695 | 0.1004 | 0.1004 | 0.2007 |
| factor(Multiplicity)2 | 0.944 | 0.7433 | 0.4368 | 0.8695 | 0.1004 | 0.1004 | 0.2007 |
| factor(Lymphatic\_invasion)2 | 0.84 | 0.6789 | 0.4669 | 0.4377 | 0.0806 | 0.0806 | 0.1611 |
| factor(EIC)2 | 0.92 | 0.6946 | 0.4606 | 0.8275 | 0.1127 | 0.1127 | 0.2254 |
| factor(NG)2 | 0.128 | 0.5489 | 0.4976 | -1.2548 | 0.2105 | 0.2105 | 0.4209 |
| factor(NG)3 | 0.872 | 0.3311 | 0.4706 | 1.6126 | 0.2705 | 0.2705 | 0.5409 |
| factor(HG)2 | 0.136 | 0.4653 | 0.4988 | -0.9567 | 0.1646 | 0.1646 | 0.3293 |
| factor(HG)3 | 0.848 | 0.3017 | 0.459 | 1.5155 | 0.2731 | 0.2731 | 0.5463 |
| factor(pT)2 | 0.68 | 0.3462 | 0.4758 | 0.7128 | 0.1669 | 0.1669 | 0.3338 |
| factor(pT)3 | 0.056 | 0.0295 | 0.1692 | 0.1148 | 0.0133 | 0.0133 | 0.0265 |
| factor(pT)4 | 0.008 | 0.0016 | 0.0395 | 0.0719 | 0.0032 | 0.0032 | 0.0064 |
| factor(pN)1 | 0.168 | 0.2671 | 0.4424 | -0.2639 | 0.0495 | 0.0495 | 0.0991 |
| factor(pN)2 | 0.032 | 0.0594 | 0.2364 | -0.155 | 0.0137 | 0.0137 | 0.0274 |
| factor(pN)3 | 0 | 0.0323 | 0.1768 | -Inf | 0.0162 | 0.0162 | 0.0323 |
| factor(ER)2 | 0.88 | 0.2114 | 0.4083 | 2.0492 | 0.3343 | 0.3343 | 0.6686 |
| factor(PR)2 | 0.952 | 0.28 | 0.449 | 3.1308 | 0.336 | 0.336 | 0.672 |
| factor(HER2)2 | 0.976 | 0.8176 | 0.3862 | 1.0308 | 0.0792 | 0.0792 | 0.1584 |
| factor(Ki\_67)2 | 0 | 0.0004 | 0.0198 | -Inf | 0.0002 | 0.0002 | 0.0004 |
| factor(RTx)2 | 0.136 | 0.2356 | 0.4244 | -0.2894 | 0.0498 | 0.0498 | 0.0996 |
| factor(CTx)2 | 0.072 | 0.372 | 0.4834 | -1.1559 | 0.15 | 0.15 | 0.3 |
| factor(HTx)2 | 0.856 | 0.205 | 0.4037 | 1.8468 | 0.3255 | 0.3255 | 0.651 |
| Summary of balance for matched data | | | | | | | |
| --- | --- | --- | --- | --- | --- | --- | --- |
| | Means Treated | Means Control | SD Control | Std.Mean Diff. | eCDF Med | eCDF Mean | eCDF Max |
| distance | 0.082 | 0.0819 | 0.0528 | 0.0015 | 0.0049 | 0.0066 | 0.0354 |
| AGE | 50.175 | 49.8792 | 10.8679 | 0.0261 | 0.0127 | 0.0169 | 0.0879 |
| factor(Multiplicity)1 | 0.0583 | 0.0646 | 0.246 | -0.0271 | 0.0033 | 0.0033 | 0.0065 |
| factor(Multiplicity)2 | 0.9417 | 0.9354 | 0.246 | 0.0271 | 0.0033 | 0.0033 | 0.0065 |
| factor(Lymphatic\_invasion)2 | 0.8333 | 0.8208 | 0.3839 | 0.034 | 0.0066 | 0.0066 | 0.0132 |
| factor(EIC)2 | 0.9167 | 0.9188 | 0.2735 | -0.0076 | 0.0009 | 0.0009 | 0.0017 |
| factor(NG)2 | 0.1333 | 0.1396 | 0.3469 | -0.0186 | 0.0034 | 0.0034 | 0.0068 |
| factor(NG)3 | 0.8667 | 0.8583 | 0.3491 | 0.0248 | 0.0045 | 0.0045 | 0.0089 |
| factor(HG)2 | 0.1417 | 0.1458 | 0.3533 | -0.0121 | 0.0024 | 0.0024 | 0.0048 |
| factor(HG)3 | 0.8417 | 0.8354 | 0.3712 | 0.0173 | 0.0035 | 0.0035 | 0.0069 |
| factor(pT)2 | 0.7083 | 0.6667 | 0.4719 | 0.089 | 0.0215 | 0.0215 | 0.0431 |
| factor(pT)3 | 0.025 | 0.0188 | 0.1358 | 0.0271 | 0.0031 | 0.0031 | 0.0062 |
| factor(pT)4 | 0 | 0 | 0 | 0 | 0 | 0 | 0 |
| factor(pN)1 | 0.175 | 0.1604 | 0.3674 | 0.0389 | 0.007 | 0.007 | 0.0139 |
| factor(pN)2 | 0.0333 | 0.0292 | 0.1684 | 0.0236 | 0.002 | 0.002 | 0.004 |
| factor(pN)3 | 0 | 0.0021 | 0.0456 | -Inf | 0.001 | 0.001 | 0.0021 |
| factor(ER)2 | 0.875 | 0.8688 | 0.338 | 0.0192 | 0.0034 | 0.0034 | 0.0068 |
| factor(PR)2 | 0.95 | 0.9667 | 0.1797 | -0.0777 | 0.0083 | 0.0083 | 0.0165 |
| factor(HER2)2 | 0.975 | 0.9812 | 0.1358 | -0.0407 | 0.0031 | 0.0031 | 0.0062 |
| factor(Ki\_67)2 | 0 | 0 | 0 | NaN | 0 | 0 | 0 |
| factor(RTx)2 | 0.1417 | 0.1333 | 0.3403 | 0.0242 | 0.0039 | 0.0039 | 0.0078 |
| factor(CTx)2 | 0.0667 | 0.05 | 0.2182 | 0.0642 | 0.0082 | 0.0082 | 0.0165 |
| factor(HTx)2 | 0.85 | 0.8417 | 0.3654 | 0.0236 | 0.0045 | 0.0045 | 0.009 |
†
† All variables are well balanced because all standardized mean differences are lower than 0.1,
the value commonly considered as threshold for balance checking
