# Supplementary material for: Clinical Characteristics and Prognosis of Metaplastic Breast Cancer Compared with Invasive Ductal Carcinoma: A Propensity-Matched Analysis
Source: Cancers (Basel). 2023 Mar 2;15(5):1556. doi: 10.3390/cancers15051556 (PMC10000576; doi:10.3390/cancers15051556)
Supplement: Supplementary file 1 [file cancers-15-01556-s001.zip › cancers-2169902-supplementary table S2.pdf]

Supplementary Table S2. Patient demographic and clinical characteristics after propensity score matching.

	Metaplastic N (%)	Ductal N (%)	***p*-value**
Age (years)			0.7782
Mean	50.18	50.00	
Std	11.53	10.74	
Multiplicity			0.7908
No	113 (94.17)	447 (93.51)	
Yes	7 (5.83)	31 (6.49)	
Lymphatic invasion			0.7081
No	100 (83.33)	392 (82.01)	
Yes	20 (16.67)	86 (17.99)	
EIC			0.9363
No	110 (91.67)	439 (91.84)	
Yes	10 (8.33)	39 (8.16)	
Nuclear grade			0.7731
Low	0 (0.00)	1 (0.21)	
Intermediate	16 (13.33)	67 (14.02)	
High	104 (86.67)	410 (85.77)	
Histologic grade			0.8557
Good	2 (1.67)	9 (1.88)	
Moderate	17 (14.17)	70 (14.64)	
Poor	101 (84.17)	399 (83.47)	
pT stage			0.1620
T1	32 (26.67)	151 (31.59)	
T2	85 (70.83)	318 (66.53)	
T3	3 (2.50)	9 (1.88)	
pN stage			0.5779
N0	95 (79.17)	386 (80.75)	
N1	21 (17.50)	77 (16.11)	
N2	4 (3.33)	14 (2.93)	
N3	0 (0.00)	1 (0.21)	
Estrogen receptor			0.8071
Negative	105 (87.5)	415 (86.82)	
Positive	15 (12.5)	63 (13.18)	
Progesterone receptor			**0.0324**
Negative	114 (95.00)	462 (96.65)	
Positive	6 (5.00)	16 (3.35)	
HER2			0.6252
Negative	117 (97.5)	469 (98.12)	
Positive	3 (2.5)	9 (1.88)	
Hormone therapy			0.7665
No	102 (85.00)	402 (84.10)	

Yes	18 (15.00)	76 (15.90)	
Radiation therapy			0.7923
No	17 (14.17)	64 (13.39)	
Yes	103 (85.83)	414 (86.61)	
Chemotherapy			0.4960
No	8 (6.67)	24 (5.02)	
Yes	112 (93.33)	454 (94.98)	

Abbreviations: EIC = Extensive intraductal component; pT = pathologic T stage; pN = pathologic N stage; N = number of patients
